# Three-Dimensional Printable Magnetic Hydrogels with Adjustable Stiffness and Adhesion for Magnetic Actuation and Magnetic Hyperthermia Applications

**DOI:** 10.3390/gels11010067

**Published:** 2025-01-15

**Authors:** Xueting Xuan, Yi Li, Xing Xu, Zhouyi Pan, Yu Li, Yonghao Luo, Li Sun

**Affiliations:** 1 School of Health Science and Engineering, University of Shanghai for Science and Technology, Shanghai 200093, China; xxt3188@gmail.com; 2 Nanotechnology Research Institute, College of Materials and Textile Engineering, Jiaxing University, Jiaxing 314001, China; liyi@zjxu.edu.cn (Y.L.);; 3 School of Materials Science and Intelligent Engineering, Nanjing University, Suzhou 215163, China; 4 Key Laboratory for Green Processing of Chemical Engineering of Xinjiang Bingtuan, School of Chemistry and Chemical Engineering, Shihezi University, Shihezi 832004, China; 5 Department of Mechanical and Aerospace Engineering, University of Houston, Houston, TX 77204, USA

**Keywords:** multi-responsive hydrogel, magnetic hydrogel, 3D-printed hydrogel structures

## Abstract

Stimuli-responsive hydrogels hold immense promise for biomedical applications, but conventional gelation processes often struggle to achieve the precision and complexity required for advanced functionalities such as soft robotics, targeted drug delivery, and tissue engineering. This study introduces a class of 3D-printable magnetic hydrogels with tunable stiffness, adhesion, and magnetic responsiveness, prepared through a simple and efficient “one-pot” method. This approach enables precise control over the hydrogel’s mechanical properties, with an elastic modulus ranging from 43 kPa to 277 kPa, tensile strength from 93 kPa to 421 kPa, and toughness from 243 kJ/m^3^ to 1400 kJ/m^3^, achieved by modulating the concentrations of acrylamide (AM) and Fe_3_O_4_ nanoparticles. These hydrogels exhibit rapid heating under an alternating magnetic field, reaching 44.4 °C within 600 s at 15 wt%, demonstrating the potential for use in mild magnetic hyperthermia. Furthermore, the integration of Fe_3_O_4_ nanoparticles and nanoclay into the AM precursor optimizes the rheological properties and ensures high printability, enabling the fabrication of complex, high-fidelity structures through extrusion-based 3D printing. Compared to existing magnetic hydrogels, our 3D-printable platform uniquely combines adjustable mechanical properties, strong adhesion, and multifunctionality, offering enhanced capabilities for use in magnetic actuation and hyperthermia in biomedical applications. This advancement marks a significant step toward the scalable production of next-generation intelligent hydrogels for precision medicine and bioengineering.

## 1. Introduction

Hydrogels have garnered significant attention in the biomedical field due to their unique three-dimensional (3D) network structure, high water content, and biocompatibility. These materials have been utilized in a variety of applications, including drug delivery systems, wound dressings, and tissue engineering scaffolds [1,2,3,4,5]. Among these, intelligent hydrogels with stimuli-responsive properties, such as temperature, pH, light, and magnetic responsiveness, have emerged as a particularly promising class of materials, with high versatility for biomedical applications.

One recent development in hydrogel application is the advent of magnetic hydrogels, which are created by incorporating magnetic nanoparticles into the hydrogel matrix. This integration endows the hydrogels with the ability to respond to external magnetic fields, enabling functionalities like targeted actuation, spatial manipulation, and magnetic hyperthermia [6]. These properties position magnetic hydrogels at the forefront of cutting-edge biomedical innovations, with applications spanning drug delivery systems [7,8], where precise localization and controlled release are critical; soft robotics [9,10], where their flexibility and responsiveness are harnessed for motion; flexible sensors [11,12], which leverage their tunable properties for detecting environmental changes; tissue engineering [13,14], where they provide scaffolds with enhanced mechanical and functional characteristics; and adsorption/separation technologies, which utilize their magnetic tunability for effective material recovery [15,16].

Magnetic hydrogels are typically composed of magnetic nanoparticles (e.g., Fe_3_O_4_) and a polymer matrix (e.g., polyacrylamide (PAM)) [2]. Currently, the primary methods for preparing magnetic hydrogels include the blending method [17], the in situ coprecipitation method [18], and the grafting method [19]. Among these, the blending method involves directly mixing magnetic nanoparticles into the hydrogel precursor. While this approach is simple and convenient, the nanoparticles tend to diffuse out of the hydrogel, leading to an uneven distribution within the gel matrix. In contrast, the in situ coprecipitation method immerses a cross-linked hydrogel into a solution containing Fe^3^⁺ and Fe^2^⁺ ions, utilizing the hydrogel’s swelling properties to synthesize magnetic particles within the gel. This method achieves uniform dispersion of the magnetic particles within the gel network; however, unreacted small molecules may compromise the biocompatibility of the magnetic hydrogel. The grafting method involves modifying the surface of magnetic nanoparticles with functional groups, but its preparation process is complex and time-consuming.

The performance of magnetic hydrogels can be tailored by modifying the properties of the hydrogel matrix (e.g., polymer structure, concentration, and degree of cross-linking) or by altering the characteristics (e.g., concentration, size, and size distribution) of the magnetic nanoparticles (MNPs) [20]. Since most cells and biological tissues are anisotropic, researchers can employ external magnetic fields to adjust the distribution of MNPs within the hydrogel network, enabling the design of anisotropic structures for cultured cells and natural tissues [21]. Currently, 3D printing technology [22,23], magnetic field-induced assembly [24], and microfluidic techniques [25] are widely used to fabricate magnetic hydrogels with ordered structures, with 3D printing being the most prominent among them. This technique not only enables the preparation of magnetic hydrogels with complex architectures but also integrates magnetic field-induced assembly during the printing process. However, 3D printing technology demands high precision in regards to both the equipment and processes. While magnetic field-induced assembly can reversibly construct magnetic materials with ordered structures, it requires the application of an external magnetic field. Moreover, the resulting structure is influenced by the magnetic field strength, as well as by the concentration and size of the MNPs. Microfluidic technology, on the other hand, offers precise control over the structure and enables the fabrication of nanoscale gel architectures, but the preparation process remains highly intricate.

Magnetic hydrogels are soft materials capable of responding to external magnetic fields, with magnetic field-induced motion being one of their fundamental characteristics. Leveraging the advantages of remote, non-contact actuation and minimal penetration depth limitations, magnetic hydrogels have garnered increasing attention in recent years. Wang et al. developed a magnetic hydrogel embedded with hard magnetic NdFeB particles, exhibiting good mechanical properties and rapid actuation under an external magnetic field [23]. Through programmed magnetization, the hydrogel achieves fast, reversible shape transformations, presenting a novel approach to controlling hydrogel shape changes via magnetic fields, distinct from previous light-driven methods. This technology holds promise for regulating more complex soft structures in the future. Huang et al. fabricated a bionic four-legged soft-film micro-robot with various controllable motion modes [26]. This micro-robot demonstrates not only the ability to overcome obstacles but also the capacity to grasp, transport, and release tiny objects by manipulating an external magnetic field. Such micro-robots, equipped with multi-modal motion and grasping capabilities, hold significant potential for biomedical applications, including stomach biopsies.

Compared to traditional cancer treatments, such as surgery, radiotherapy, and chemotherapy, hyperthermia represents an emerging adjuvant therapy with promising potential. Tumor hyperthermia typically falls into two categories based on temperature windows. The first method involves heating tumors to temperatures above 50 °C, effectively killing cancer cells rapidly. However, such high temperatures can cause collateral damage to surrounding normal tissues, leading to certain side effects [27]. Mild hyperthermia, on the other hand, involves heating specific tissues or organs to temperatures between 41 °C and 46 °C [28]. Within this range, protein and DNA denaturation render tumor cells more prone to apoptosis or increase their sensitivity to radiation and anti-cancer drugs [29]. Nevertheless, mild hyperthermia alone is insufficient for complete tumor eradication and often requires its combination with other therapeutic modalities to enhance its efficacy. Magnetic hyperthermia leverages the heat generation capability of magnetic nanoparticles (MNPs) under an alternating magnetic field. This technique offers several advantages, including non-invasiveness, high biological safety, precise targeting, and the ability to locally and deeply heat tissues [30,31]. Fe_3_O_4_ nanoparticles, known for their unique magnetic properties and biocompatibility, generate heat when exposed to varying magnetic fields. Incorporating these nanoparticles into hydrogel matrices allows for localized temperature increases in targeted tissues via external magnetic fields, inducing cell damage or death. This method has been validated as an effective approach for cancer treatment. Wang et al. developed a magnetic dual-network (M-DN) hydrogel with superior mechanical properties (high modulus of 0.4 MPa and toughness of 1500 J/m^2^), ion-resistance stability, and rapid magnetic induction heating via an in situ co-precipitation method. This magnetic gel is applicable for tissue hyperthermia therapy and drug delivery through magnetic induction heating [31]. Similarly, Wu et al. created an injectable, self-healing magnetic colloidal hydrogel (MCH) for minimally invasive hepatocellular carcinoma treatment via ultrasound-guided magnetic high-temperature ablation [32]. This innovation presents a new strategy for ablating deep tumors with minimal invasiveness. Magnetic hydrogels, with their good magnetic, mechanical, and biocompatible properties, enable precise temperature control during hyperthermia treatment, further enhancing their therapeutic potential [33].

This research presents an effort to develop a versatile 3D-printable magnetic hydrogel platform that uniquely combines tunable mechanical properties, adhesion, and multifunctionality for advanced biomedical applications. Unlike traditional magnetic hydrogels, which often lack precise control over their mechanical and rheological characteristics, our hydrogels achieve adjustable stiffness (elastic modulus ranging from 43 kPa to 277 kPa), tensile strength (93 kPa to 421 kPa), and toughness (243 kJ/m^3^ to 1400 kJ/m^3^) through a simple “one-pot” synthesis method. Additionally, the incorporation of nanoclay and Fe_3_O_4_ nanoparticles ensures high structural fidelity and printability, enabling the fabrication of complex 3D geometries with good reproducibility. The hydrogels also exhibit controlled heating under an alternating magnetic field, reaching 44.4 °C in 600 s, which is highly favorable for applications in magnetic hyperthermia. These performance metrics, combined with the efficient and scalable synthesis approach, indicate significant advancement in this work over previous efforts, addressing practical usability and broadening the scope of applications for magnetic hydrogels in precision medicine and bioengineering.

## 2. Results and Discussion

### 2.1. Preparation and Characterization of PAM-Fe_3_O_4_ Magnetic Hydrogels

**Figure 1:**
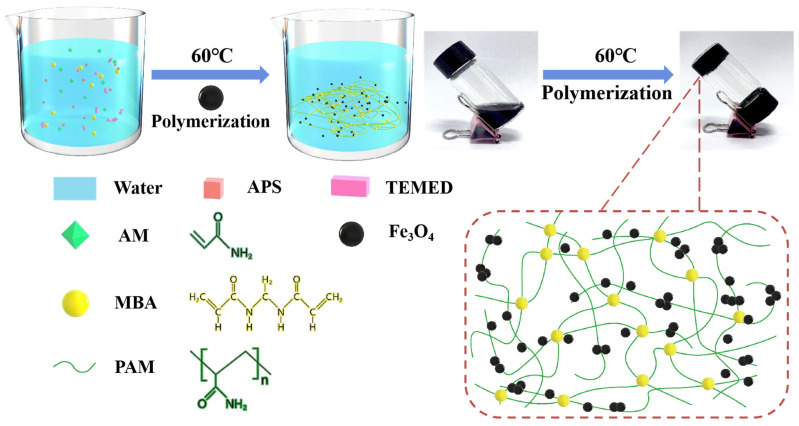
Schematics of the evolution of PAM-Fe_3_O_4_ magnetic hydrogel formation.

The blending method is the most commonly used approach for preparing magnetic hydrogels due to its simplicity [13]. In this work, the PAM-Fe_3_O_4_ magnetic hydrogel was prepared using a simple “one-pot” method (also known as the blending method), as shown in Figure 1. First, the AM monomer, MBA cross-linking agent, APS initiator, and TEMED catalytic accelerator were successively added into a beaker containing DI water, and the PAM solution was obtained by stirring the mixture evenly. The chemical gelation process is exothermic, during which APS will be thermally degraded into sulfate anion radicals, and protons are extracted from the water molecules to produce hydroxyl radicals [34,35]. These radicals attach to and activate the AM monomers, and under the action of the TEMED catalyst, these free radicals initiate AM monomers and polymerize to form linear polymer chains Second, the Fe_3_O_4_ dispersion solution dispersed by ultrasound for 20 min was added to the PAM solution for mixing. To promote PAM chemical polymerization, a processing temperature of 60 °C was used in preparing the gel samples. At this temperature, these linear polymer chains finally form chemically cross-linked PAM gel in the presence of MBA as a cross-linker. Due to the hydroxyl and carboxyl groups on the surface of Fe_3_O_4_, the nanoparticles bond to the cross-linked PAM network through hydrogen bonding. Additionally, the Fe_3_O_4_ nanoparticles are uniformly dispersed throughout the cross-linked PAM network, resulting in the formation of the PAM-Fe_3_O_4_ magnetic hydrogel.

**Figure 2:**
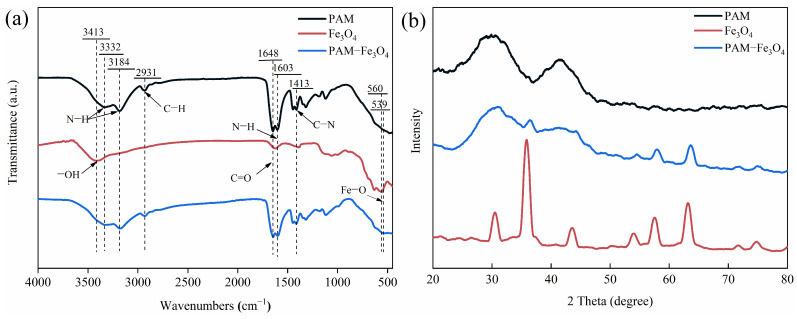
(**a**) FT-IR spectra of PAM hydrogels, Fe_3_O_4_, and PAM-Fe_3_O_4_ magnetic hydrogels; (**b**) XRD patterns of PAM hydrogels, Fe_3_O_4_, and PAM-Fe_3_O_4_ magnetic hydrogels.

To investigate the molecular interaction between Fe_3_O_4_ and PAM, FT-IR spectroscopic analysis was conducted. Figure 2a shows the FT-IR spectra from the PAM, Fe_3_O_4_, and PAM-Fe_3_O_4_ magnetic hydrogel samples. The absorption characteristic peak of free and associative –NH_2_ in PAM appears at 3332 cm^−1^ and 3184 cm^−1^, respectively [14]. The characteristic peak of amide II (N–H bending vibration) is at 1603 cm^−1^. The peak corresponds to the C–H stretching vibration located at 2931 cm^−1^. The characteristic absorption peak of the carbonyl group occurs at 1648 cm^−1^. The C–N stretching vibration peak appears at 1413 cm^−1^. The –OH characteristic peak of the Fe_3_O_4_ is located at 3413 cm^−1^ [36], and the characteristic peaks of Fe–O and –COOH are located at 560 cm^−1^ and 1623 cm^−1^, respectively [18,32,37]. This indicates that there are certain hydroxyl and carboxyl groups on the surface of Fe_3_O_4_ particles. Compared to the Fe_3_O_4_ and PAM samples, characteristic FT-IR peaks of the PAM-Fe_3_O_4_ magnetic hydrogel samples all exhibit red shifts; these indicate that the peaks corresponding to the N–H bonds of PAM at 3332 cm^−1^, 3184 cm^−1^, and 1603 cm^−1^ shift to 3324 cm^−1^, 3180 cm^−1^, and 1601 cm^−1^ for the PAM-Fe_3_O_4_ magnetic hydrogel. The absorption characteristic peak of amide I shifted from 1648 cm^−1^ to 1644 cm^−1^. Meanwhile, the Fe–O characteristic peak of Fe_3_O_4_ moved to 539 cm^−1^ from 560 cm^−1^. The shift in the characteristic peaks indicates that there is an electrostatic interaction between Fe_3_O_4_ and the polymer chains [14], and the hydroxyl and carboxyl groups on the surface of the nanoparticles form hydrogen bonds with the amino groups on the PAM polymer chains [36]. The C–N characteristic peak at 1413 cm^−1^ indicates that PAM-Fe_3_O_4_ magnetic hydrogel is composed of -CONH_2_. The peak at 539 cm^−1^ is attributed to the Fe–O stretching vibration [36].

Figure 2b shows the XRD patterns of the PAM, Fe_3_O_4_, and PAM-Fe_3_O_4_ magnetic hydrogels. The Fe_3_O_4_ nanoparticles display some distinct peaks at 2 θ values of 30.4°, 35.8°, 43.5°, 53.9°, 57.5°, and 63.2°, assigned to the (220), (311), (400), (422), (511), and (440) planes of the cubic spinel crystalline of Fe_3_O_4_ (Powder Diffraction file, JCPDS card no. 19–0629), respectively. The XRD pattern of the PAM-Fe_3_O_4_ magnetic hydrogel showed the characteristic diffraction peak of Fe_3_O_4_, which indicated that the PAM-Fe_3_O_4_ magnetic hydrogel was successfully synthesized [16]. In addition, the strength of the characteristic diffraction peaks in magnetic hydrogels is weakened by the interaction between the polymer chain and Fe_3_O_4_ [36].

**Figure 3:**
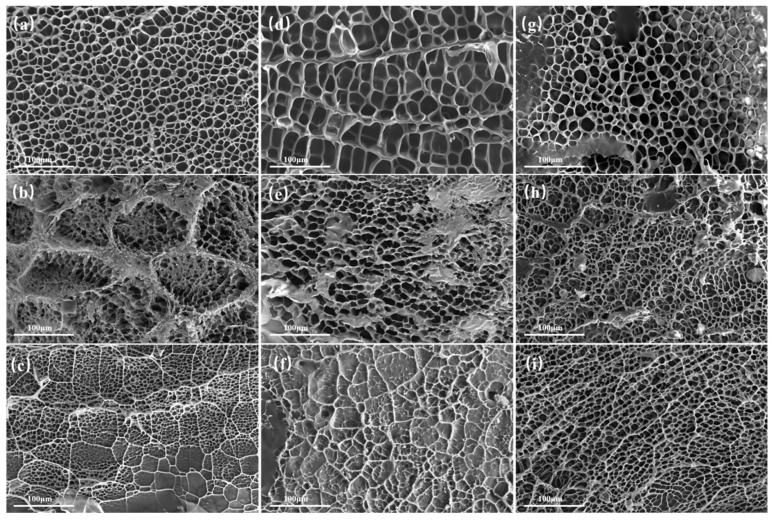
SEM images of PAM and PAM-Fe_3_O_4_ magnetic hydrogels with different concentrations. (**a**–**c**) SEM of 13.8 wt% PAM and 13.8 wt% PAM magnetic hydrogels; (**d**–**f**) SEM images of 18.4 wt% PAM and 18.4 wt% PAM magnetic hydrogels; (**g**–**i**) SEM images of 23 wt% PAM and 23 wt% PAM magnetic hydrogels.

SEM images of the cross-sections of the swollen hydrogels are presented in Figure 3. After freeze-drying, all hydrogel samples exhibit a porous cross-sectional structure. Figure 3a, Figure 3d, and Figure 3g show the SEM images of 13.8 wt%, 18.4 wt%, and 23 wt% PAM, respectively. The gel structure is evenly distributed, and the pore walls are smooth. When 5 wt% Fe_3_O_4_ is added to PAM, Fe_3_O_4_ nanoparticles adsorb onto the molecular chains of PAM, resulting in smaller pores in the PAM network, as shown in Figure 3b,e,h. Figure 3c,f,i show SEM images of hydrogels with the addition of 10 wt% Fe_3_O_4_, where the pore size of the magnetic gel gradually decreases as the Fe_3_O_4_ concentration increases. Figure S1 displays the SEM and TEM images of the Fe_3_O_4_ magnetic nanoparticles. These magnetic nanoparticles are spherical, with an average size of approximately 10 nm. Additionally, the dynamic light scattering results (Figure S2) show that the particle size of the Fe_3_O_4_ dispersion is about 50 nm after ultrasonic treatment for 20 min, and the dispersion system is stable.

### 2.2. Mechanical Properties of PAM-Fe_3_O_4_ Magnetic Hydrogels

**Figure 4:**
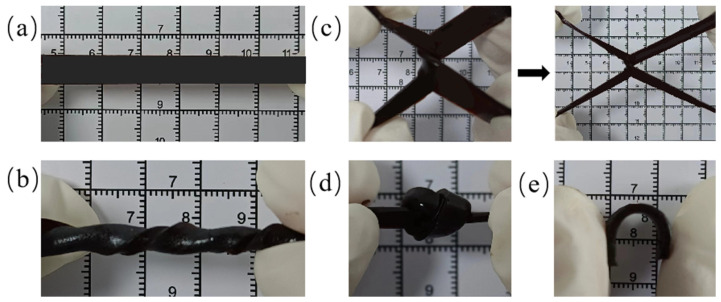
Mechanical behavior of the PAM-Fe_3_O_4_ magnetic hydrogels: (**a**) stretching, (**b**) twisting, (**c**) cross-stretching, (**d**) knotting, and (**e**) bending.

PAM-Fe_3_O_4_ magnetic hydrogels exhibit improved elasticity and strength compared to those of pure PAM hydrogels. As illustrated in Figure 4, these hydrogels also demonstrate good ductility, allowing them to endure significant tensile deformation both in their original state and after crossing (Figure 4a,c). Moreover, PAM-Fe_3_O_4_ magnetic hydrogels can exhibit various mechanical behaviors, such as twisting (Figure 4b), knotting (Figure 4d), and bending (Figure 4e).

Swelling tests were conducted on the hydrogel samples, and the calculated swelling ratios for PAM-Fe_3_O_4_ magnetic hydrogels with varying AM and Fe_3_O_4_ concentrations are presented in Figure S3. The swelling ratio of the PAM hydrogels decreased with a reduction in the AM mass fraction. Specifically, the swelling ratios for PAM with 23 wt%, 18.4 wt%, and 13.8 wt% AM were 363.8%, 316.9%, and 290.2%, respectively. Additionally, the swelling ratios of magnetic gels with different concentrations diminished after 50 h of swelling. At a constant PAM concentration, the swelling ratio of the magnetic gels decreased with an increase in Fe_3_O_4_ content. For example, in 23 wt% PAM, the swelling ratio decreased from 300.9% to 277.7% as the Fe_3_O_4_ content increased from 5 wt% to 15 wt%. This reduction in the swelling ratio is likely due to the presence of Fe_3_O_4_ nanoparticles, which act as rigid particles occupying part of the PAM network space. These particles hinder the extension of polymer chains during swelling, thereby reducing the swelling capacity of the gel [38].

**Figure 5:**
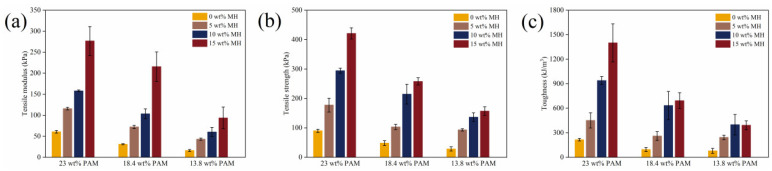
Tensile properties of PAM and PAM-Fe_3_O_4_ magnetic hydrogels with different concentrations. (**a**) Tensile modulus; (**b**) tensile strength; (**c**) toughness.

Quasistatic tensile and compression tests were conducted to evaluate the mechanical properties of the pure PAM and PAM-Fe_3_O_4_ hydrogels with Fe_3_O_4_ concentrations ranging from 0 wt% to 15 wt%. As shown in Figure 5, the tensile modulus, strength, and toughness of pure PAM decreased with a reduction in the AM mass fraction. This is because a lower AM mass fraction results in a looser gel network structure, thereby reducing the mechanical properties of the gel. The addition of Fe_3_O_4_ to PAM was found to enhance the modulus, strength, and toughness of the hydrogel. This improvement can be attributed to the role of Fe_3_O_4_ as a nano-enhancer, dispersing within the gel network and restricting the movement of the polymer chains. Furthermore, the hydrogen bonds formed between the amino groups of the PAM polymer chains and the hydroxyl and carboxyl groups of Fe_3_O_4_ contribute to the enhanced mechanical properties of the magnetic gel. At a constant AM mass fraction, increasing the Fe_3_O_4_ concentration leads to a greater number of hydrogen bonds between PAM and Fe_3_O_4_, thereby further improving the mechanical properties of the magnetic gel. Notably, the 23 wt% PAM–15 wt% Fe_3_O_4_ magnetic hydrogels exhibited good mechanical properties, including a tensile modulus of 276.7 kPa, a tensile strength of 421 kPa, and a toughness of 1400 kJ/m^3^, which were significantly higher than those of 23 wt% PAM (tensile modulus of 62.5 kPa, tensile strength of 90 kPa, and toughness of 215 kJ/m^3^). Additionally, the stretching cycles for the 23 wt% PAM–10 wt% Fe_3_O_4_ magnetic hydrogels under different strains were performed, as shown in Figure S4a. Energy dissipation at this concentration increased with higher tensile strain, as illustrated in Figure S5a. During the first stretching cycle, as shown in Figures S4b and S5b, a significant number of hydrogen bonds between PAM and Fe_3_O_4_ were broken. In subsequent cycles, the stress–strain curves overlapped, indicating the magnetic gel’s good recovery characteristics. To investigate the effect of different AM mass fractions on the stretching cycle of magnetic gels at a constant Fe_3_O_4_ concentration, Figures S4c and S5c reveal that the energy dissipation of magnetic gels decreased as the AM mass fraction decreased.

**Figure 6:**
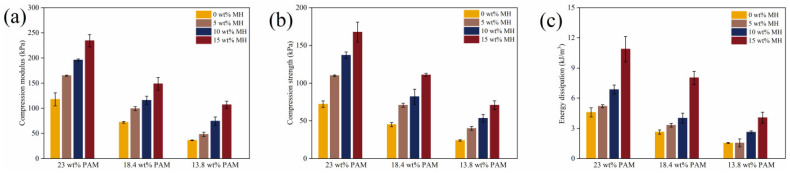
Compression properties of PAM and PAM-Fe_3_O_4_ magnetic hydrogels with different concentrations. (**a**) Compressive modulus; (**b**) compressive strength; (**c**) energy dissipation of one compression cycle under 50% strain.

Figure 6 illustrates the compression properties of PAM and PAM-Fe_3_O_4_ magnetic hydrogels. As described previously, the compression modulus and compressive strength of magnetic hydrogels increase with higher Fe_3_O_4_ concentrations, as shown in Figure 6a,b. The energy dissipation of PAM and PAM-Fe_3_O_4_ magnetic hydrogels under 50% strain during one compression cycle is presented in Figure 6c. The results indicate that the energy dissipation of magnetic hydrogels rises with an increase in Fe_3_O_4_ concentration. This phenomenon can be attributed to Fe_3_O_4_ acting as a nano-filler, forming more hydrogen bonds with PAM that break during the compression cycle, thereby increasing energy dissipation. The stress–strain curves and energy dissipation behavior of 23 wt% PAM–10 wt% Fe_3_O_4_ magnetic hydrogels under different compressive strains are shown in Figure S6. These findings align with the experimental results from the tensile cycles, demonstrating that the energy dissipation of magnetic hydrogels increases as compressive strain intensifies.

### 2.3. Rheological Characterizations of PAM-Fe_3_O_4_ Magnetic Hydrogels

Laponite nanoclay, when dispersed in water, forms a physical gel due to its unique ability to self-assemble into a “card house” structure. This structure arises from electrostatic interactions between the positively charged edges and the negatively charged faces of the Laponite platelets. These interactions create a dynamic, interconnected network that provides the material with gel-like properties, such as high viscosity and yield stress, even at low concentrations. This “card house” structure is a hallmark of physical gels, characterized by its reversibility and sensitivity to external forces.

The shear-thinning behavior of this nanoclay gel is particularly advantageous for 3D printing. Under applied shear stress, such as during extrusion through a nozzle, the “card house” structure collapses as the Laponite platelets realign in the direction of the flow. This structural collapse significantly reduces the viscosity, allowing the material to flow smoothly and extrude easily. Once the shear force is removed, the platelets spontaneously reassemble into the “card house” configuration, restoring the gel’s viscosity and mechanical stability. This reversible transition ensures that the printed material retains its shape and fidelity, enabling the fabrication of complex geometries with high precision.

By incorporating Laponite nanoclay into hydrogel precursors, the shear-thinning effect facilitates controlled material deposition, while the post-extrusion recovery of the gel network enhances the mechanical integrity of the printed structure. This dual functionality makes nanoclay-enhanced hydrogels highly suitable for extrusion-based 3D printing, paving the way for the creation of robust, intricately designed constructs for advanced applications in tissue engineering, soft robotics, and other biomedical fields.

**Figure 7:**
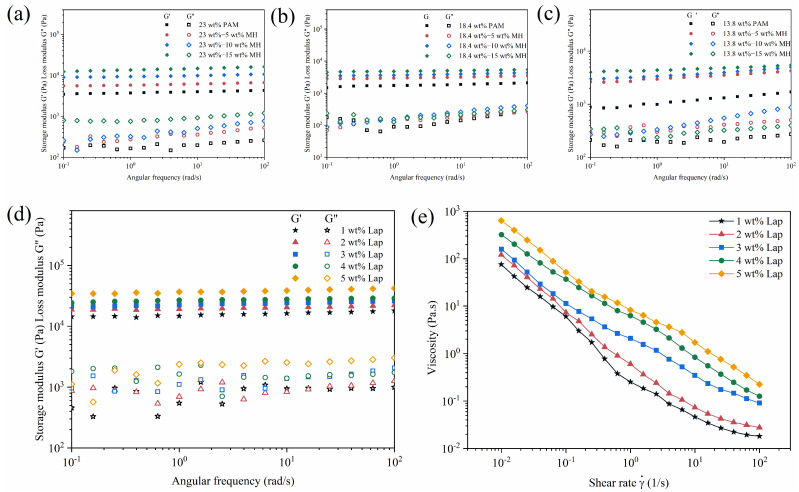
Frequency scanning of PAM, PAM–Fe_3_O_4_, and PAM–Fe_3_O_4_–Laponite hydrogels with different concentrations. (**a**–**c**) Frequency scanning of 23 wt%, 18.4 wt%, 13.8 wt% PAM and PAM–Fe_3_O_4_ magnetic hydrogels. (**d**,**e**) Frequency scanning of 23 wt% PAM–10 wt% Fe_3_O_4_ gel and flow scanning of precursor gel with different concentrations of nanoclay.

Figure 7 illustrates the frequency scanning results of the PAM, PAM-Fe_3_O_4_, and PAM-Fe_3_O_4_-Laponite hydrogels with varying concentrations. From Figure 7a–c, it is evident that the storage modulus (G’) of pure PAM decreases as the AM mass fraction decreases. This can be attributed to the lower AM mass fraction, leading to reduced cross-linking density, a looser gel network structure, and consequently, a lower elastic modulus. Additionally, Figure 7a–c demonstrate that at the same PAM mass fraction, G’ increases with higher Fe_3_O_4_ concentrations. FT-IR analysis suggests that this is due to specific interactions between PAM and Fe_3_O_4_. Figure 7d reveals that the storage modulus (G’) of the PAM-Fe_3_O_4_-Laponite hydrogels increases with greater Laponite nanoclay content. This enhancement is likely due to hydrogen bonding between the nanoclay and PAM [39,40], which strengthens the mechanical properties of the hydrogel. The flow scanning results (Figure 7e) indicate that the precursor gel exhibits shear-thinning behavior with increasing shear rate. This shear-thinning characteristic enhances the extrudability of the precursor material, enabling the layer-by-layer fabrication of three-dimensional structures with adequate rigidity [41].

### 2.4. Adhesion and Magnetic Properties of PAM-Fe_3_O_4_ Magnetic Hydrogels

**Figure 8:**
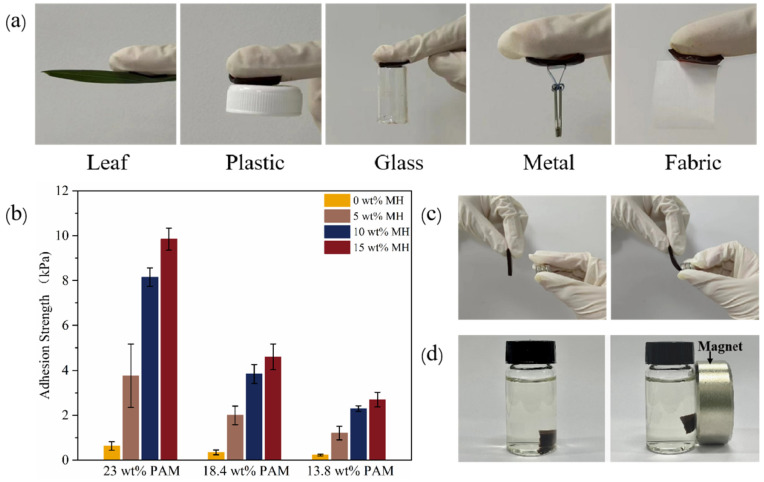
(**a**) Photographs displaying the PAM-Fe_3_O_4_ magnetic hydrogel adhering different objects. (**b**) Adhesion strength of different magnetic hydrogels on glass substrates at room temperature. (**c**,**d**) Photographs of the PAM-Fe_3_O_4_ magnetic hydrogel interacting magnets in air and water.

The adhesion properties of hydrogels are critical for their effectiveness in biomedical applications such as tissue engineering, wound healing, and drug delivery [42,43,44]. The adhesion characteristics of gels are influenced by several key factors, including the presence of functional groups of the polymer, surface energy and wettability, hydration level and interface morphology, surface roughness, etc. As shown in Figure 8a, prepared PAM and PAM-Fe_3_O_4_ gels exhibit good adhesion to various materials such as natural materials, polymers, glasses, metals, and fabrics. The interplay of physical and chemical interactions between the gel and the substrate governs the adhesion performance [45,46,47,48]. Among these, hydrogen bonding plays a pivotal role in adhesion, particularly on hydrophilic surfaces [47]. For example, glass surfaces are hydrophilic and rich in hydroxyl (-OH) groups. The amide (-CONH_2_) groups in PAM can form hydrogen bonds with these hydroxyl groups, facilitating strong adhesion. Figure 8b shows the adhesion strength of PAM and PAM-Fe_3_O_4_ magnetic hydrogels with different concentrations on the glass substrate. The addition of Fe_3_O_4_ nanoparticles to PAM will further enhance this interaction due to the introduction of additional hydrogen bonding sites or electrostatic interactions with the negatively charged glass surface. In contrast, most metal surfaces are covered with a thin layer of oxide or hydroxide, which promotes the formation of hydrogen bonds with the amide groups in PAM. Fe_3_O_4_ nanoparticles can interact by forming strong hydrogen bonds with metal surface hydroxyl groups and through ionic interactions. Additionally, the Fe_3_O_4_ nanoparticles can establish coordination bonds with metal ions in the oxide layer, further improving adhesion. Figure 8c,d show images of the magnetic hydrogels in air, water and induced by a magnet. The results show that the magnetic hydrogel displays good magnetic responsiveness and can move flexibly under the action of an external magnetic field. See Video S1 for the video images of the magnetic driving of the magnetic hydrogel under glassware containing water.

**Figure 9:**
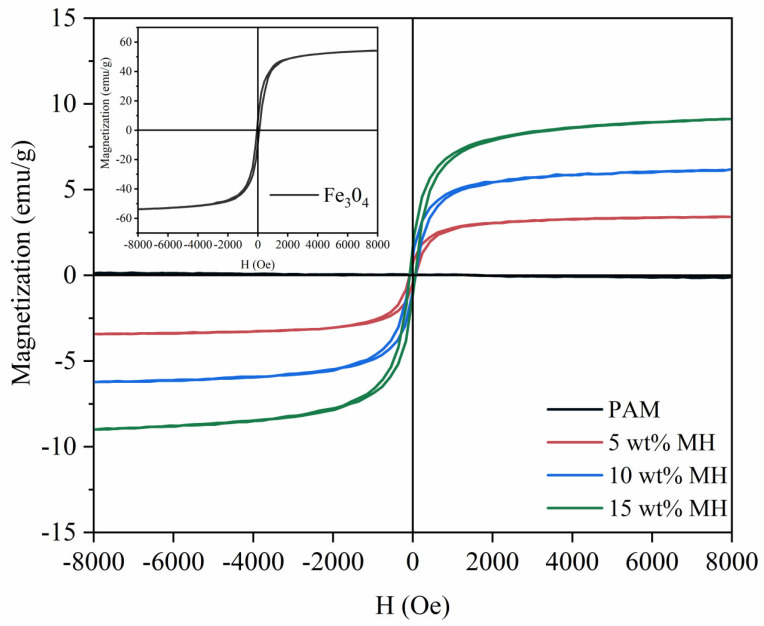
Room-temperature magnetic hysteresis loop of the PAM and PAM−Fe_3_O_4_ hydrogels; the inset shows the magnetization curve of the Fe_3_O_4_ nanoparticles.

To further evaluate the magnetic properties of the hydrogels, the hysteresis loops of the Fe_3_O_4_, PAM, and PAM-Fe_3_O_4_ magnetic hydrogels were measured using a vibrating sample magnetometer, as shown in Figure 9. The hysteresis loop of Fe_3_O_4_ at room temperature exhibits a saturation magnetization of 54 emu/g and a coercivity of only 79 Oe. PAM, as expected, exhibits diamagnetic properties. For the PAM-Fe_3_O_4_ magnetic hydrogels, the saturation magnetization values for 5 wt%, 10 wt%, and 15 wt% Fe_3_O_4_ content are 3.41 emu/g, 6.15 emu/g, and 9.05 emu/g, respectively. Although the magnetic gel exhibits relatively weak magnetization compared to that of pure Fe_3_O_4_, the hysteresis loops remain narrow, demonstrating low coercivity. As the content of magnetic particles increases, the saturation magnetization of the hydrogels also gradually increases. This indicates that the simplest and most effective way to enhance the magnetic properties of magnetic hydrogels is to increase the Fe_3_O_4_ content [17].

In an alternating magnetic field, magnetic iron oxide nanoparticles absorb external magnetic energy and convert it into thermal energy. Magnetic hyperthermia utilizes this phenomenon to transfer the generated heat to surrounding lesion tissues, inducing thermal damage and achieving therapeutic effects. Currently, the heat generation mechanisms of magnetic materials in an alternating field are believed to primarily include eddy currents, hysteresis, and relaxation [28,49]. Under the influence of an alternating magnetic field, the magnetic gel absorbs energy and converts it into heat, generating a magnetocaloric effect [31]. Since the conductivity of Fe_3_O_4_ magnetic particles is very low, eddy current-induced heat can be ignored [28,50]. Therefore, this discussion focuses mainly on the hysteresis and relaxation heat generation mechanisms of magnetic nanoparticles. Under the influence of the external magnetic field, the hysteresis and relaxation of Fe_3_O_4_ nanoparticles generate heat. The Néel relaxation time is obtained using Formula (1), where *τ_0_* is the time constant (~10^−9^ s), *K* is the anisotropy constant, *V* is the volume of the magnetic nanoparticle, *k* is the Boltzmann constant, and *T* is the absolute temperature. The power loss corresponding to the Néel relaxation is approximated using Formula (2), where *m* is the magnetic moment of the particle, *H* is the amplitude of the magnetic field, *ω* is the measured angular frequency, and *ρ* is the density of magnetite. Therefore, the Néel relaxation time is related to various parameters of the magnetic nanoparticles, and the power can be adjusted by changing the intensity or frequency of the magnetic field, thus adjusting the temperature change of the magnetic heat of the magnetic hydrogel [50].

(1) $$\tau_{N}=\tau_{0}\;exp\frac{KV}{kT}$$

*P* = (*mHωτ_N_*)^2^/[2*τ_N_kTρV*(1 + *ω^2^τ_N_*^2^)] (2)

**Figure 10:**
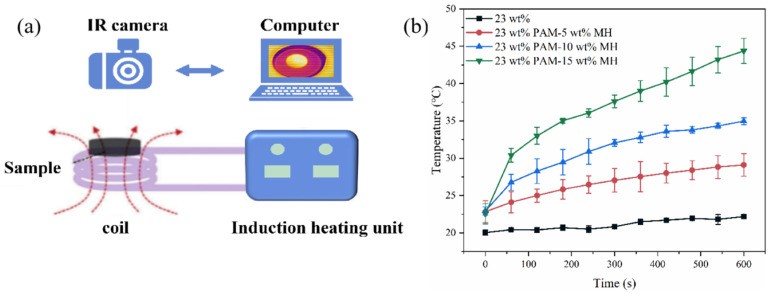
Magnetic hyperthermia effect of magnetic hydrogels. (**a**) The experimental setup diagram of magnetic hyperthermia includes an infrared camera, a computer, and an induction heating unit with an alternating magnetic field. (**b**) The surface temperature of the PAM and PAM-Fe_3_O_4_ hydrogels as a function of heating time.

Figure 10 illustrates the magnetothermal effects of 23 wt% PAM and the corresponding magnetic hydrogels at different Fe_3_O_4_ concentrations. When the Fe_3_O_4_ nanoparticles are embedded in the gel structure, the magnetic moments of the nanoparticles align with the AMF. During this process, as the external magnetic field changes direction, the internal magnetic moment generates heat. Additionally, the magnetic particles themselves rotate and generate heat when their orientation relative to the external magnetic field changes. These phenomena are described by the Néel and Brown effects [28,49], and the heat produced by these effects increases the temperature of the magnetic hydrogel.

As shown in Figure 10a, the magnetic hydrogel is heated under the influence of AMF. Gel samples with different concentrations were placed in a coil and exposed to an AMF for 10 min, starting at an initial temperature of about 22 °C. The average temperature of the 5–15 wt% Fe_3_O_4_ magnetic gels increased to approximately 29.1 °C, 35 °C, and 44.4 °C, respectively. However, in reality, the normal body temperature is 37 °C, and the temperature range suitable for mild hyperthermia is between 41 °C and 46 °C. This range can induce cytotoxicity in cancerous tissues. When the temperature exceeds 46 °C, tumor cells undergo necrosis, leading to cell death [29]. While this may enhance tumor cell ablation, it could potentially damage surrounding healthy tissues [31]. Therefore, the magnetothermal temperature of the 15 wt% PAM-Fe_3_O_4_ magnetic gel designed in this study is appropriate for mild magnetic hyperthermia. Furthermore, the results indicate that for a fixed AM mass fraction of the magnetic gel, the magnetothermal effect becomes more pronounced with increasing Fe_3_O_4_ content. The concentration of Fe_3_O_4_ is a critical factor in determining the magnetothermal effect of the gel [51]. Thus, the heating temperature generated by the magnetothermal effect can be adjusted by controlling the Fe_3_O_4_ content or the strength of the magnetic field.

### 2.5. Three-Dimensional Printing of PAM-Fe_3_O_4_ Magnetic Hydrogel

**Figure 11:**
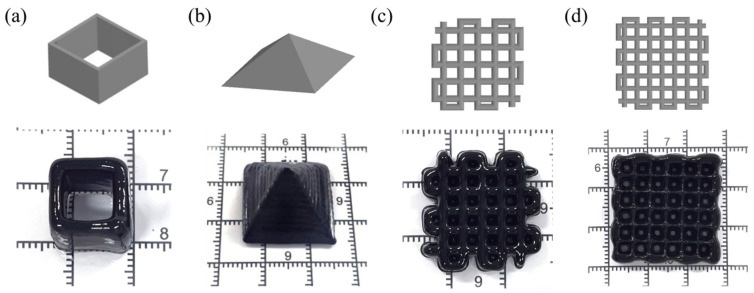
Examples of several 3D-printed 23 wt% PAM–10 wt% Fe_3_O_4_–5 wt% Laponite magnetic hydrogel structures: (**a**) square frame; (**b**) triangular pyramid; (**c**,**d**) mesh structure.

Viscoelasticity is crucial for the printability of the hydrogel precursor. Due to its unique rheological properties, Laponite nanoclay is a material that undergoes sol–gel transformation under shear stress [52]. As a result, it is commonly employed to enhance the viscoelasticity of extruded 3D printing precursors [53,54]. The 3D printing process of the PAM-Fe_3_O_4_ magnetic hydrogel is shown in Video S2. Figure 11 illustrates several 3D-printed structures, such as square frames, triangular pyramids, and 90° grid structures, made from a 23 wt% PAM–10 wt% Fe_3_O_4_ precursor with 5 wt% Laponite nanoclay. These 3D-printed structures demonstrate good shape fidelity.

While 3D-printable magnetic hydrogels offer significant promise for applications such as magnetic actuation and hyperthermia, potential biocompatibility and material stability challenges still exist for practical application. Possible leaching of nanoparticles out of the hydrogel matrix over time can potentially reduce the material’s magnetic responsiveness and raise biocompatibility issues [55]. Strategies to mitigate this include surface modification of the nanoparticles to promote their interaction with the polymer matrix or to enhance polymer cross-linking to securely embed nanoparticles within the gel network [55,56,57]. Another challenge is ensuring long-term material stability, as repeated mechanical loading, magnetic excitation, or prolonged exposure to physiological conditions may degrade the hydrogel’s structural integrity and functionality. Incorporating reinforcing agents such as nanoclays or optimizing the cross-linking density can enhance the hydrogel’s durability and resistance to environmental stressors. Furthermore, developing hydrogels with self-healing capabilities or using protective coatings could help maintain their performance over extended periods. Addressing these challenges will be critical to advancing the practical utility and reliability of magnetic hydrogels in biomedical and engineering applications.

## 3. Conclusions

In this study, PAM-Fe_3_O_4_ magnetic hydrogels with adjustable stiffness, adhesion, and mild magnetic hyperthermia capability were prepared using a simple “one-pot” method. Furthermore, the hydrogel precursor was modified with nanoclay to enhance its rheological properties, making it suitable for extrusion-based 3D printing, which enables the production of precise and complex hydrogel structures. The uniform distribution of low-concentration magnetic particles in the hydrogel was achieved via a simple “one-pot” method using magnetic dispersion. For a 23 wt% PAM–15 wt% Fe_3_O_4_ hydrogel sample, the measured tensile modulus, tensile strength, and toughness can reach 277 kPa, 421 kPa, and 1400 kJ/m^3^, respectively. The enhancement of the gel’s mechanical properties is attributed to the hydrogen bonds formed between the PAM polymer chain and the Fe_3_O_4_ surface. Such samples exhibit fast mechanical actuation responses under magnetic fields. Mild magnetic hyperthermia can be achieved and easily tuned by choosing the Fe_3_O_4_ particle type and concentration and excitation field characterization. Additionally, the introduction of nanoclay as a rheological modifier and the shear-thinning effect of the hydrogel precursor enable the successful 3D printing of magnetic hydrogels. It is envisioned that such a 3D-printable hydrogel, with good adhesion, compatible mechanical properties, magnetic field response, and magnetic hypothermia capability, can broaden biomedical applications.

By further tuning the hydrogel network structure, e.g., the polymer composition, cross-link density, and magnetic nanoparticle content, it is possible to modulate their responsiveness to external magnetic fields, control their swelling behavior, and optimize their mechanical stiffness or elasticity. For drug delivery, precisely calibrated gel architectures can enable stimuli-responsive release profiles, delivering therapeutic molecules directly to targeted tissues upon the application of a magnetic field. Meanwhile, in tissue engineering, the gels’ magnetically controllable properties allow for dynamic mechanical cues and spatially directed cell growth, enhancing cell proliferation and tissue formation. As a result, tailored magnetic hydrogels provide a versatile platform that can be adapted to specific design parameters in diverse biomedical applications.

## 4. Materials and Methods

### 4.1. Materials

Acrylamide (AM, AR 99%), N,N,N’,N’-tetramethyl ethylenediamine (TEMED), and 25 wt% Fe_3_O_4_ dispersion (Fe_3_O_4_ particle size: 10–30 nm, average particle size 20 nm) were purchased from Macklin Biochemical Co., Ltd., Shanghai, China. N,N’-Methylenebisacrylamide (MBA) and ammonium peroxydisulphate (APS) were obtained from Aladdin Co., Ltd., Shanghai, China. Synthetic hectorite Laponite-XLG (chemical formula: [Mg_5.34_Li_0.66_Si_8_O_20_(OH)_4_]Na_0.66_) was purchased from Guangzhou Daixun Trading Co., Ltd., Guangzhou, China. The average exfoliated nanoclay particle size is about 25 nm in diameter and 1 nm in thickness. Deionized (DI) water was used in all experiments.

### 4.2. Preparation of PAM-Fe_3_O_4_ Hydrogels

The PAM-Fe_3_O_4_ magnetic hydrogels were prepared using the “one-pot” method. First, the monomer AM (6.9 g), the cross-linker MBA (15.3 mg) (0.08 mol% to AM), the initiator APS (12.9 mg) (0.06 mol% to AM), and the catalyst accelerator TEMED (22.2 μL) (0.17 mol% to AM) were added to DI water under stirring. A certain amount of Fe_3_O_4_ dispersion (25 wt%) was added to the above mixed solution after ultrasonic dispersion for 20 min. The solution was poured into a mold and polymerized at 60 °C for 1 h to form a hydrogel. The mass fraction of Fe_3_O_4_ ranged from 5–15 wt% (relative to the total weight of the polymer and water), and magnetic gels with different concentrations were named 5–15 wt% MH. The compositions of the samples are shown in Table S1.

### 4.3. Characterization

#### 4.3.1. Infrared Spectroscopy

FT-IR spectra from the samples were recorded using a Thermo Scientific Nicolet iS20 infrared spectrometer (Waltham, MA, USA) in the wavenumber range of 4000–400 cm^−1^. The 25 wt% Fe_3_O_4_ dispersion was centrifuged and dried to obtain Fe_3_O_4_ particles. Fe_3_O_4_ powder samples were prepared by grinding with KBr and measured in the transmission mode, and the PAM and PAM-Fe_3_O_4_ magnetic hydrogel samples were measured in the attenuated total reflection (ATR) mode after freeze-drying.

#### 4.3.2. X-Ray Diffraction

The copper target was used to conduct the XRD measurement of Fe_3_O_4_, PAM and PAM-Fe_3_O_4_ magnetic hydrogel at the scanning speed of 5°/min and within the range of 20–80° using the German Bruker D8 Advance diffractometer (Billerica, MA, USA).

#### 4.3.3. Observation of Hydrogel Morphology

Microstructures of the PAM-Fe_3_O_4_ magnetic hydrogels were examined using a scanning electron microscope (SEM, Gemini SEM 300, Oberkochen, Baden-Wurttemberg, Germany) at an acceleration voltage of 15 kV. The PAM-Fe_3_O_4_ magnetic hydrogel samples were first quenched in liquid nitrogen and fractured before freeze-drying for 48 h. The PAM-Fe_3_O_4_ magnetic hydrogel cross-section samples were coated with a thin layer of gold for SEM analyses. In addition, the morphology of the 25 wt% Fe_3_O_4_ dispersion was observed using SEM and TEM, respectively. The 25 wt% Fe_3_O_4_ dispersion was first sonicated and dried, followed by gold spraying. The sample was tested using a field emission scanning electron microscope (Thermo Fisher Scientific Apreo 2S+, Thermo Fisher Scientific, Waltham, MA, USA) at an acceleration voltage of 10 kV. After a small amount of the 25 wt% Fe_3_O_4_ dispersion sample was dispersed uniformly by ultrasound, 1–2 drops were taken from the dropper onto the copper grid, and the floating liquid was absorbed by filter paper, which was left to stand. After the sample was dried, it was photographed by a transmission electron microscope (TEM, FEI Tecnai F20, FEI Company, Hillsboro, OR, USA) under a voltage of 200 kV.

#### 4.3.4. Swelling Testing of PAM and PAM-Fe_3_O_4_ Hydrogels

The prepared PAM, 5–15 wt% PAM-Fe_3_O_4_ magnetic hydrogels were immersed in DI water at room temperature and weighed at regular time intervals until swelling equilibrium was reached. The swelling ratio was calculated using the following formula: swelling ratio (SR) = (W_s_ − W_d_)/W_d_ × 100%, where Ws is the mass of the swollen hydrogels and W_d_ is the mass of the original sample.

#### 4.3.5. Dynamic Light Scattering

The Zeta potential and particle size of the 25 wt% Fe_3_O_4_ dispersion were measured by Malvern Zetasizer Nano ZS90 (Malvern Panalytical, Malvern, Worcestershire, UK) after 20 min of ultrasound exposure.

#### 4.3.6. Mechanical Performances of PAM and PAM-Fe_3_O_4_ Hydrogels

Both tensile and compression tests were performed on a CMT2502 universal test machine (Sansi Taijie Electrical Equipment Co., Ltd., Zhuhai, China). The size of the tensile test specimens was 4 mm in width, 1–2 mm in thickness, and 34 mm in length between the clamps. The elastic modulus E was calculated from the stress–strain curve slope using the data between 10–15% strain. Two types of tensile cyclic testing were performed, i.e., one with gradually increasing maximum tensile strain from 10% to 400%, and the other repeated at a maximum strain of 400% for 10 times. In the compression test, the hydrogel samples had a cylindrical (8 mm in height and 20 mm in diameter) shape. The compression cyclic tests included a series of sequentially increasing maximum compression strains from 15% to 70% and the other repeated at a maximum strain of 50% only once. The 180° peeling test of PAM and PAM-Fe_3_O_4_ magnetic gel with different concentrations was carried out using a glass plate. The sample width is 5 mm, and the adhesive length is 10 mm. The stretching rate for all tensile tests is 100 mm/min. The peeling speed of the adhesion test and the tensile rate of the compression test are 5 mm/min. The compression strength is defined as the stress when the compression strain reached 50%. The dissipated energy is calculated from the enclosed area between the loading–unloading curves. The adhesion strength is defined as the maximum force received divided by the area. Each test was repeated for at least three samples.

#### 4.3.7. Rheological Testing of PAM and PAM-Fe_3_O_4_ Hydrogels

The viscoelastic behavior of the PAM, PAM-Fe_3_O_4_, and PAM-Fe_3_O_4_-Laponite hydrogels was tested at room temperature using the 25 mm parallel plates with a DHR-2 rheometer produced by TA Instruments Company (New Castle, DL, USA). The storage modulus (G’) and loss modulus (G”) of the different hydrogel samples were determined in the frequency range from 0.1 to 100 rad/s; the strain amplitude was 0.1%. Flow scanning was performed on the precursor of 23 wt% PAM–10 wt% Fe_3_O_4_ Laponite at a shear rate of 0.01–100 S^−1^ and a frequency of 10 rad/s. The concentration of Laponite added to the precursor ranged from 1 wt% to 5 wt%. All tests were performed at 25 °C.

#### 4.3.8. Vibrating Sample Magnetometry (VSM)

The 25 wt% Fe_3_O_4_ dispersion was centrifuged and dried to obtain Fe_3_O_4_ particles. After freeze-drying 18.4 wt% PAM and PAM-Fe_3_O_4_ magnetic gel for 12 h, the magnetic hysteresis loops of the Fe_3_O_4_ particles and hydrogels were measured using a vibrating sample magnetometer (VSM) (Lakeshore 8604, Westerville, OH, USA) at room temperature, with a maximum magnetic field amplitude of 8 kOe, where M is the magnetization, and H is the applied magnetic field. After weighing each sample, the respective magnetization response was given in emu/g.

#### 4.3.9. Magnetic Induction Heating

The PAM-Fe_3_O_4_ magnetic hydrogels were heated under an alternating magnetic field (AMF). The AMF setup consists of a power supply, a water-cooling system, and an induction heating system (copper tube resonant frequency: 76 kHz; maximum current: 28 A; DC voltage: 53.5 V). The solenoid used for heating has 8 turns, with a diameter of 50 mm. An infrared (IR) camera (HIKMICRO TPK20, Hikvision, Hangzhou, China) was used to capture the thermal images.

#### 4.3.10. Three-Dimensional Printing of PAM-Fe_3_O_4_ Magnetic Hydrogels

Extrusion printing of the 23 wt% PAM–10 wt% Fe_3_O_4_–5 wt% Laponite hydrogel precursor was performed using a 3D printer (Bio-Architect SR, Hangzhou Regenovo Bio-Tech Co., Ltd., Hangzhou, China). The printed precursor was converted into a hydrogel by heating at 60 °C. A printing needle with an inner diameter of 0.41 mm was selected, and a square frame, a triangular pyramid, and a 90° grid structure were printed at a printing pressure of 0.1 MPa and a printing speed of 15 mm/s. The number of square frames and triangular pyramids is 20 layers, and the model sizes are 10 mm × 10 mm and 20 mm × 20mm, respectively. The two different shapes of the grid are printed with 15 and 10 layers, the model sizes are 20 mm × 20 mm and 25 mm × 25 mm, and the fill spacings are 3.5 mm and 4 mm, respectively. The 3D printing parameters of PAM-Fe_3_O_4_ magnetic hydrogel are shown in Table S2.

## Data Availability

The original contributions presented in this study are included in the article/Supplementary Materials. Further inquiries can be directed to the corresponding authors.

## Supplementary Materials

The following supporting information can be downloaded at: https://www.mdpi.com/article/10.3390/gels11010067/s1, Figure S1: The SEM images of (a,b) SEM images of 25 wt% Fe_3_O_4_ dispersion, (c,d) TEM images of 25 wt% Fe_3_O_4_ dispersion; Figure S2: (a) Particle size distribution of Fe_3_O_4_ in 25 wt% Fe_3_O_4_ dispersion, (b) Zeta potential of 25 wt% Fe_3_O_4_ dispersion; Figure S3: The swelling ratio versus time curves of PAM-Fe_3_O_4_ magnetic hydrogels with different concentrations at different times; Figure S4: Tensile cycling experiment of PAM-Fe_3_O_4_ magnetic hydrogels. (a) Tensile cycling experiments of 23 wt% PAM-10 wt% MH under different strains, (b) tensile cycling experiment of 23 wt% PAM-10 wt% MH at 400% strain for 10 times, (c) 23 wt% PAM-10 wt% MH, 18.4 wt% PAM-10 wt% MH, 13.8 wt% PAM-10 wt% MH were cycled once at 400% tensile strain; Figure S5: Energy dissipation of the stretching cycle of PAM-Fe_3_O_4_ magnetic hydrogels. (a) Energy dissipation of 23 wt% PAM-10 wt% MH during stretching cycles under different strains, (b) energy dissipation test of 23 wt% PAM-10 wt% MH after 10 stretching cycles at 400% strain, (c) energy dissipation of 23 wt% PAM-10 wt% MH, 18.4 wt% PAM-10 wt% MH, 13.8 wt% PAM-10 wt% MH after one cycle at 400% tensile strain, Figure S6. Compression cycle of PAM-Fe_3_O_4_ magnetic hydrogels. (a) Compression cycles of 23 wt% PAM-10 wt% MH under different strains, (b) energy dissipation of 23 wt% PAM-10 wt% MH during compression cycle under different strains; Table S1: The composition of PAM-Fe_3_O_4_ magnetic hydrogels; Table S2: 3D printing parameters of PAM-Fe_3_O_4_ magnetic hydrogels, Video S1: The magnetic driving of the magnetic hydrogel, Video S2: The 3D printing process of the magnetic hydrogel.
