# Supplementary material for: Three-Dimensional Printable Magnetic Hydrogels with Adjustable Stiffness and Adhesion for Magnetic Actuation and Magnetic Hyperthermia Applications
Source: Gels. 2025 Jan 15;11(1):67. doi: 10.3390/gels11010067 (PMC11764729; doi:10.3390/gels11010067)
Supplement: Supplementary file 1 [file gels-11-00067-s001.zip › gels-3367641 Supporting Information.pdf]

# Supporting Information

## 3D printable magnetic hydrogels with adjustable stiffness and adhesion for magnetic actuation and magnetic hyperthermia applications

**Xueting Xuan [a], Yi Li [b], Xing Xu [c], Zhouyi Pan [b], Yu Li [b], Yonghao Luo [d]*, and Li Sun [e]****

[a] School of Health Science and Engineering, University of Shanghai for Science and Technology, Shanghai 200093, China

[b] College of Materials and Textile Engineering & Nanotechnology Research Institute, Jiaxing University, Jiaxing 314001, China

[c] School of Materials Science and Intelligent Engineering, Nanjing University, Suzhou 215163, China

[d] School of Chemistry and Chemical Engineering, Key Laboratory for Green Processing of Chemical Engineering of Xinjiang Bingtuan, Shihezi University, Shihezi 832004, China

[e] Department of Mechanical and Aerospace Engineering, University of Houston, Houston, Texas 77204, USA

*Corresponding author: Y. H. Luo and L. Sun

Address: University of Houston, Houston, Texas, USA

Telephone: 7137434509

Email: lsun4@uh.edu (L.Sun) and luoyonghao@dlut.edu.cn (Y. H. Luo)

**Table S1.** The composition of PAM-$Fe_3O_4$ magnetic hydrogels.

| Hydrogels | AM （g） | $Fe_3O_4$ + Water （g） | MBA （g） | APS （g） | TEMED （μL） | Total （g） |
|---|---|---|---|---|---|---|
| 23 wt% PAM | | 0 + 23 | | | | |
| 23 wt% PAM-5 wt% $Fe_3O_4$ | | 6 + 17 | | | | |
| 23 wt% PAM-10 wt% $Fe_3O_4$ | 6.9 | 12 + 11 | 0.0153 | 0.0129 | 22.2 | 30 |
| 23 wt% PAM-15 wt% $Fe_3O_4$ | | 18 + 5 | | | | |
| 18.4 wt% PAM | | 0 + 30.5 | | | | |
| 18.4 wt% PAM-5 wt% $Fe_3O_4$ | | 7.5 + 23 | | | | |
| 18.4 wt% PAM-10 wt% $Fe_3O_4$ | 6.9 | 15 + 15.5 | 0.0153 | 0.0129 | 22.2 | 37.5 |
| 18.4 wt% PAM-15 wt% $Fe_3O_4$ | | 22.5 + 8 | | | | |
| 13.8 wt% PAM | | 0 + 43 | | | | |
| 13.8 wt% PAM-5 wt% $Fe_3O_4$ | | 10 + 33 | | | | |
| 13.8 wt% PAM-10 wt% $Fe_3O_4$ | 6.9 | 20 + 23 | 0.0153 | 0.0129 | 22.2 | 50 |
| 13.8 wt% PAM-15 wt% $Fe_3O_4$ | | 30 + 13 | | | | |

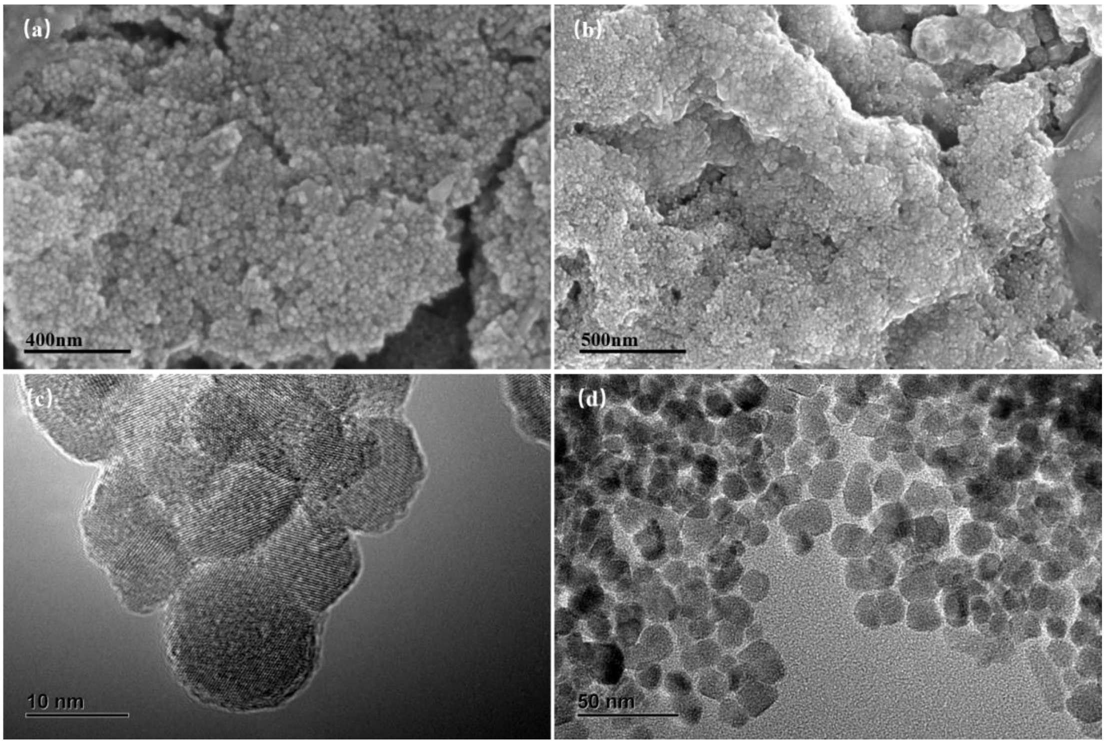


**Figure S1.** The SEM images of (a-b) SEM images of 25 wt% $Fe_3O_4$ dispersion, (c-d) TEM images of 25 wt% $Fe_3O_4$ dispersion.

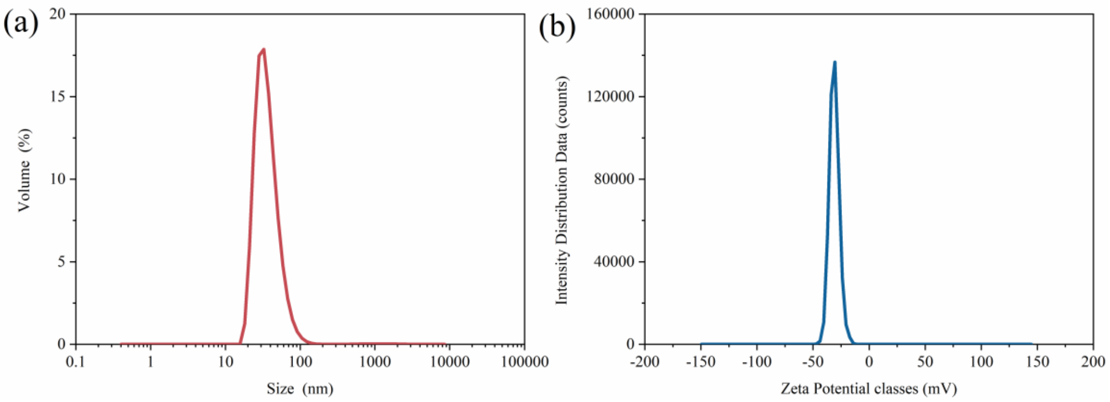


**Figure S2.** (a) Particle size distribution of $Fe_3O_4$ in 25 wt% $Fe_3O_4$ dispersion, (b) Zeta potential of 25 wt% $Fe_3O_4$ dispersion.

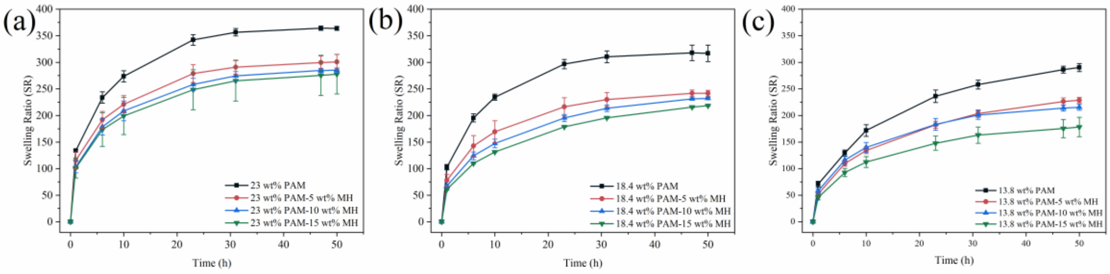


**Figure S3.** The swelling ratio versus time curves of PAM-$Fe_3O_4$ magnetic hydrogels with different concentrations at different times.

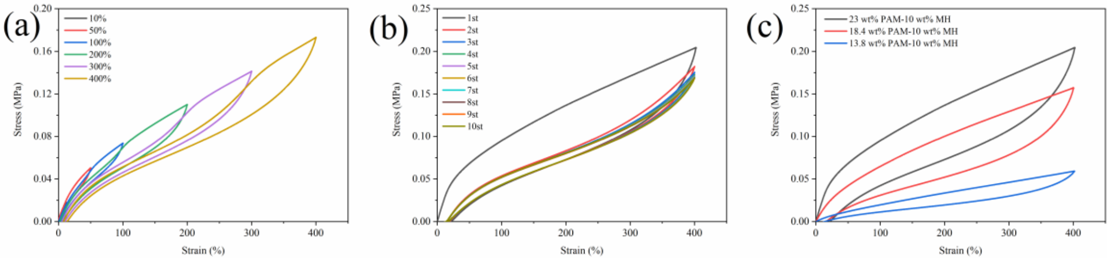


**Figure S4.** Tensile cycling experiment of PAM-$Fe_3O_4$ magnetic hydrogels. (a) Tensile cycling experiments of 23 wt% PAM-10 wt% MH under different strains, (b) tensile cycling experiment of 23 wt% PAM-10 wt% MH at 400% strain for 10 times, (c) 23 wt% PAM-10 wt% MH, 18.4 wt% PAM-10 wt% MH, 13.8 wt% PAM-10 wt% MH were cycled once at 400% tensile strain.

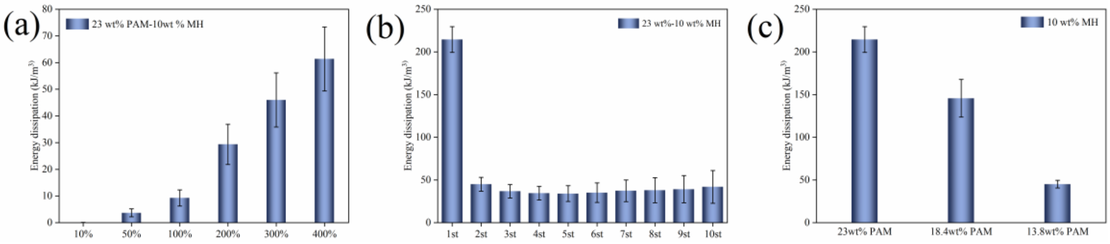


**Figure S5.** Energy dissipation of the stretching cycle of PAM-$Fe_3O_4$ magnetic hydrogels. (a) Energy dissipation of 23 wt% PAM-10 wt% MH during stretching

cycles under different strains, (b) energy dissipation test of 23 wt% PAM-10 wt% MH after 10 stretching cycles at 400% strain, (c) energy dissipation of 23 wt% PAM-10 wt% MH, 18.4 wt% PAM-10 wt% MH, 13.8 wt% PAM-10 wt% MH after one cycle at 400% tensile strain.

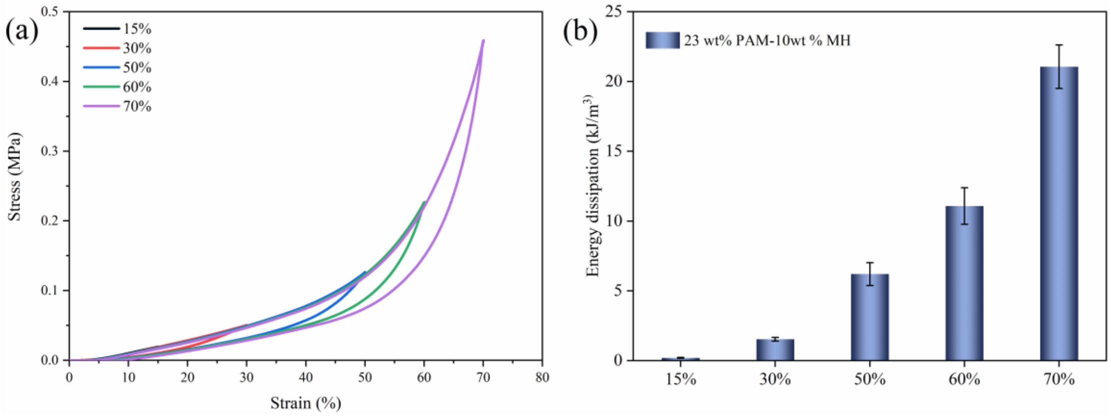


**Figure S6.** Compression cycle of PAM-$Fe_3O_4$ magnetic hydrogels. (a) Compression cycles of 23 wt% PAM-10 wt% MH under different strains, (b) energy dissipation of 23 wt% PAM-10 wt% MH during compression cycle under different strains.

**Table S2.** 3D printing parameters of PAM-$Fe_3O_4$ magnetic hydrogels.

| Hydrogel structures | Printing needle | Printing pressure | Printing speed | Model size | Printing layers |
|---|---|---|---|---|---|
| Square frame | | | | 10 mm × 10 mm | 20 layers |
| Triangular pyramid | inner diameter of 0.41 mm | 0.1 MPa | 15 mm/s | 20 mm × 20 mm | 20 layers |
| mesh structure | | | | 20 mm × 20 mm | 15 layers |
| | | | | 25 mm × 25 mm | 10 layers |
